# Human Air-Liquid-Interface Organotypic Airway Cultures Express Significantly More ACE2 Receptor Protein and Are More Susceptible to HCoV-NL63 Infection than Monolayer Cultures of Primary Respiratory Epithelial Cells

**DOI:** 10.1128/spectrum.01639-22

**Published:** 2022-07-12

**Authors:** Gino Castillo, Juan Carlos Mora-Díaz, Rahul K. Nelli, Luis G. Giménez-Lirola

**Affiliations:** a Department of Veterinary Diagnostic and Production Animal Medicine, College of Veterinary Medicine, Iowa State University, Ames, Iowa, USA; University of Siena

**Keywords:** ACE2, Alphacoronavirus, coronavirus, HCoV-NL63, infection, LLC-MK2, organotypic airway cultures, primary human respiratory epithelial cells, upper respiratory tract

## Abstract

Human coronavirus NL63 (HCoV-NL63) is commonly associated with mild respiratory tract infections in infants, being that the respiratory epithelial cells are the main target for infection and initial replication of this virus. Standard immortalized cells are highly permissive to HCoV-NL63, and they are routinely used for isolation and propagation of the virus from clinical specimens. However, these cell lines are not the natural cell target of the virus and lack sufficient complexity to mimic the natural infection process *in vivo*. This study comparatively evaluated the differences on the susceptibility to HCoV-NL63 infection and virus replication efficiency of submerged monolayer cultures of LLC-MK2 and primary human respiratory epithelial cells (HRECs) and organotypic airway cultures of respiratory cells (ALI-HRECs). Productive viral infection and growth kinetics were assessed by morphologic examination of cytopathic effects, immunofluorescence, reverse transcription quantitative real-time PCR, and flow cytometry. Results from this study showed higher susceptibility to HCoV-NL63 infection and replication in LLC-MK2 cells followed by ALI-HRECs, with very low susceptibility and no significant virus replication in HRECs. This susceptibility was associated with the expression levels of angiontensin-converting enzyme 2 (ACE2) receptor protein in LLC-MK2, ALI-HRECs, and HRECs, respectively. Remarkably, organotypic ALI-HREC cultures expressed significantly more ACE2 receptor protein and were more susceptible to HCoV-NL63 infection than monolayer cultures of HREC. The ACE2 receptor is, therefore, a critical factor for susceptibility to HCoV-NL63 infection and replication, as is the type of culture used during infection studies.

**IMPORTANCE** HCoV-NL63 is widespread globally, accounting for a significant number of respiratory infections in children and adults. HCoV-NL63 gains entrance into respiratory epithelial cells via the ACE2 receptor, the same cell receptor used by severe acute respiratory syndrome coronavirus (SARS-CoV) and SARS-CoV-2. Thus, HCoV-NL63 has been suggested as safe surrogate for studying disease mechanisms and therapeutic interventions against SARS-like CoVs, while working under BSL-2 conditions. The present study not only showed the critical role of ACE2 for effective HCoV-NL63 infection and replication, but also shed light on the need of more refined and complex *in vitro* organotypic models that recapitulate the proxy of air-liquid respiratory epithelia cell composition, structure, and functionality. These cultures have broaden virological studies toward improving our understanding of how coronaviruses cause disease and transmission not just within humans but also in animal populations.

## INTRODUCTION

Human coronavirus NL63 (HCoV-NL63) belongs to the subfamily *Orthocoronavirinae* and genus *Alphacoronavirus* (1). HCoV-NL63 was first isolated in 2003 in tertiary monkey kidney cells from a 7-month-old child suffering from bronchiolitis and conjunctivitis (2). HCoV-NL63 is predominantly associated with upper and lower respiratory tract infections in young children (3). Mild respiratory symptoms include fever, rhinorrhea, pharyngitis, cough, and sore throat, while more serious lower respiratory tract symptoms are croup, bronchitis, bronchiolitis, and/or pneumonia (4–8).

Different studies suggest that HCoV-NL63 is widespread globally, accounting for a significant number of children hospitalizations (9, 10). Nevertheless, syndromic diagnosis of HCoV-NL63-associated disease is difficult because it relies on clinical symptoms, which are nonspecific and overlap with those commonly reported in other viral and bacterial respiratory infections. Virus isolation from clinical specimens in cell culture remains the most reliable evidence for confirmation of infection and virus viability (11). There are several cell lines derived from the kidneys, lungs, or intestines that are permissive to HCoV-NL63. Those include LLC-MK2, Vero (B4, E6, FM), MRC-5, Caco-2, tertiary monkey kidney cells, renal proximal tubule epithelial cells, human renal epithelial cells, and primary human airway epithelial cells (2, 11–15).

Perhaps most importantly, like severe acute respiratory syndrome coronavirus (SARS-CoV) (16) and SARS-CoV-2 (17, 18), HCoV-NL63 utilizes angiotensin-converting enzyme 2 (ACE2) as its receptor for binding to viral spike (S) protein and cell entry, essential steps in establishing viral infection (19). Therefore, HCoV-NL63 has been suggested as safe BSL-2 model/surrogate for studying disease mechanisms and therapeutic interventions against SARS-like CoVs.

Often, preclinical antiviral screening relies on the detection and quantification of replicating a virus in cell culture. The virus must bind and enter efficiently to target cells; however, not all cells support viral gene expression or promote efficient viral release at levels required for efficient spread. In this study we assessed the differences on cell susceptibility and replication efficiency to HCoV-NL63 infection in LLC-MK2 cells, as standard immortalized cells routinely used for virus isolation and propagation, and primary human respiratory epithelial cells (HRECs), which is the main target for infection and replication, grown as monolayers or as organotypic air-liquid interface (ALI) cultures. Refining *in vitro* cell culture models for mild to low pathogenic common cold coronaviruses such as HCoV-NL63 will help to identify factors related to the infection and replication efficiency and to develop therapeutic interventions against current and future emerging coronaviruses.

## RESULTS

### LLC-MK2 cells are highly permissive to HCoV-NL63, allowing virus replication at high titers.

**FIG 1:**
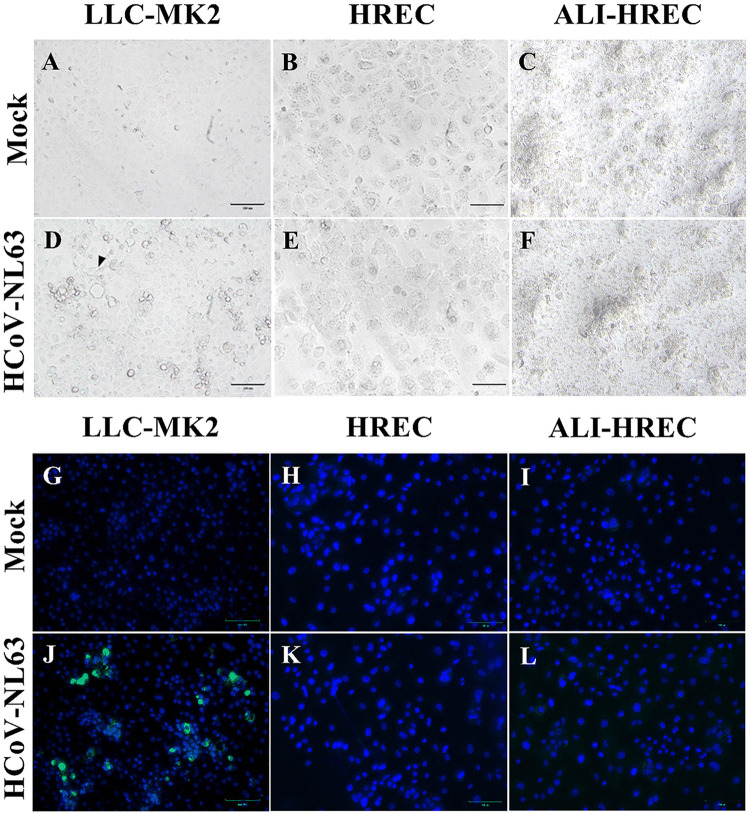
Microscopic evaluation of the susceptibility of LLC-MK2 cells and human respiratory epithelial cells (HRECs) to HCoV-NL63 infection. Confluent monolayer (submerged) cultures of LLC-MK2 cells and HRECs were inoculated with HCoV-NL63 at 1 × 10^5^ TCID_50_/mL or mock inoculated (infection medium) and incubated for 96 h (*n* = 3). (A to F) Brightfield images of LLC-MK2 and HREC cultures at 96 h postinoculation (hpi). Mock-inoculated with LLC-MK2 cells (A), HRECs (B), and ALI-HRECs (C). (D) Cytopathic effects (CPE) in HCoV-NL63-infected LLC-MK2 cells, including clumping of cells and cytoplasmic stranding with cell detachment (see arrows). (E to F) Absence of CPE in HREC and ALI-HREC cultures. (G to L) Immunofluorescence staining for HCoV-NL63 N protein (green) and DAPI nuclear counterstain (blue). The indirect immunofluorescence assay (IFA) was performed using an IgG1 mouse monoclonal anti-HCoV-NL63 N protein (2D4; Ingenasa-Eurofins) at a final concentration of 0.25 μg/mL, followed by incubation with 20 μg/mL of FITC-conjugated goat anti-mouse IgG antibody (KPL; SeraCare Life Sciences Inc.), while cell nuclei were stained using NucBlue Fixed cell ReadyProbes reagent (DAPI; Invitrogen, Thermo Fisher Scientific). (G to I) Absence of unspecific fluorescence in mock-inoculated IFA controls for LLC-MK2 cells (G), HRECs (H), and ALI-HRECs (I). (J to L) HCoV-NL63 N protein (green) was detected in LLC-MK2 cells (J) but not in HRECs (K) nor in ALI-HRECs (L). Scale bar = 100 μm.

**FIG 2:**
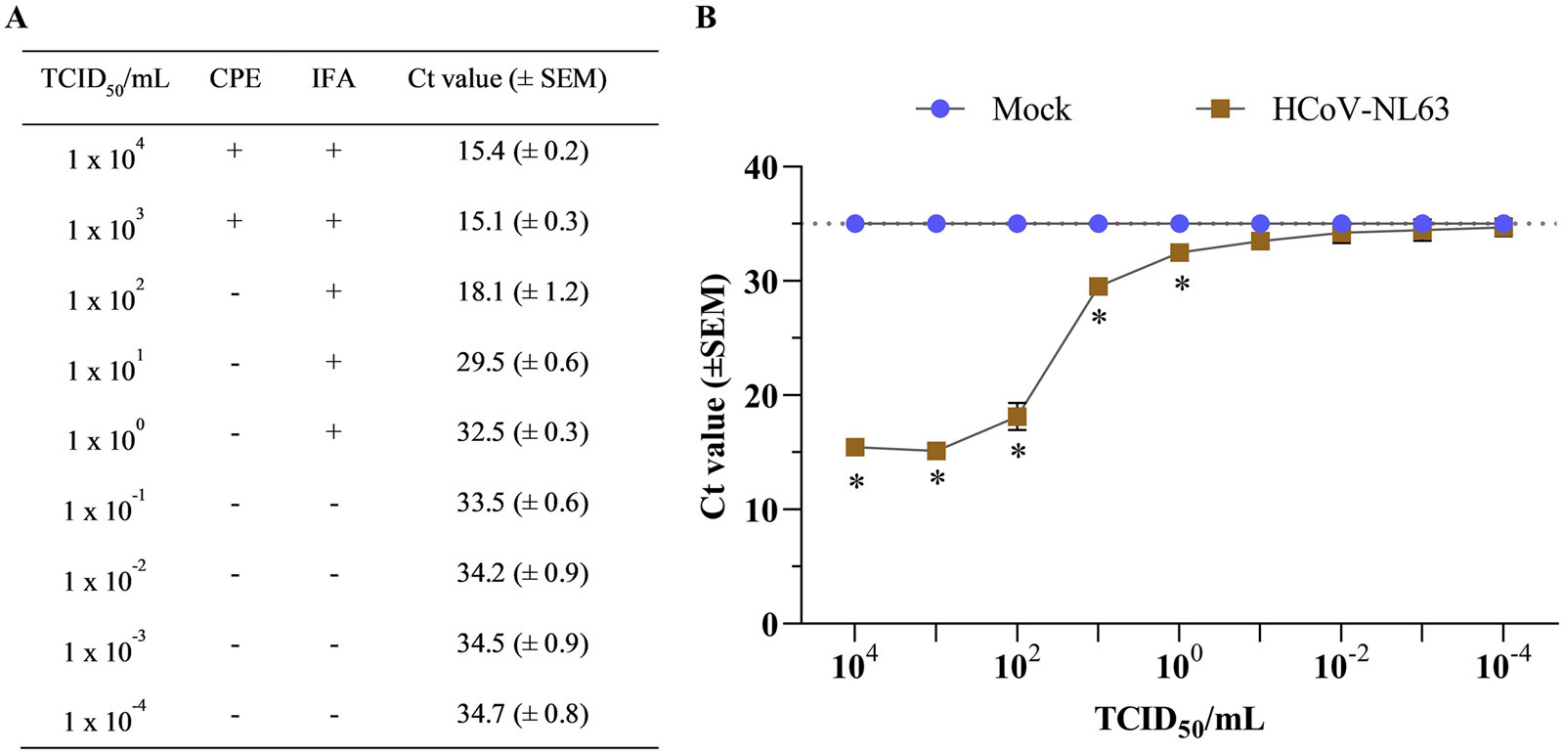
Titration HCoV-NL63 cultured in LLC-MK2 cells. (A) Comparing HCoV-NL63 detection at different viral dilutions after 120 h postinoculation (hpi) using cytopathic effects (CPE), indirect immunofluorescence assay (IFA), and real-time quantitative PCR (RT-qPCR). The presence of CPE and immunofluorescence against the HCoV-NL63 N protein is represented with a (+) sign, while the absence of infection is represented with a (−) sign. Viral RNA detection was determined using RT-qPCR and expressed as mean threshold cycle (*C_T_*) values (±standard error of the mean [SEM]) (B) Graph representing mean *C_T_* values plotted from nine 10-fold serial dilutions of HCoV-NL63 (*n* = 7)- or mock-inoculated (infection medium) LLC-MK2 cells (*n* = 1) after 120 hpi. The mean *C_T_* values were determined from collected supernatants using rRT-qPCR targeting the HCoV-NL63 nucleocapsid (N) gene. Samples with a (*C_T_*) value >35 were considered negatives. * Statistically significant difference denoted with *P* < 0.05.

Following HCoV-NL63 inoculation of fresh monolayer cultures of LLC-MK2 cells, virus-specific cytopathic effect (CPE), understood as the changes in the characteristic epithelial morphology (Fig. 1A), viability, and alter cell-cell interactions in infected cells, was observed by 72 hpi. The CPE was weak or diffuse and transient, characterized by rounded cells and cytoplasmic stranding, clump and detachment of dead cells (Fig. 1D). After the virus was harvested, the HCoV-NL63 stock titer was determined by performing 10-fold serial dilutions (10^−4^ to 10^4^ 50% tissue culture infective dose [TCID_50_]/mL) and the assessment of viral infection was based on the presence of CPE, positive fluorescent staining of HCoV-NL63 N protein by immunofluorescence assay (IFA), and viral RNA detection by reverse transcription quantitative real-time PCR (RT-qPCR) (Fig. 2A). Virus-specific CPE was only detected at high viral doses above 10^3^ TCID_50_/mL, while positive immunofluorescence staining was detected until 10° TCID_50_/mL virus infectious dose. However, the high analytical sensitivity of the HCoV-NL63 RT-qPCR detected virus infection at very low titers, with threshold cycle (*C_T_*) values ranging between 15.4 for 10^4^ TCID_50_/mL and >34.2 for TCID_50_/mL above 10^−2^ (Fig. 2B). A final virus titer of 1.18 × 10^5^ TCID_50_/mL was determined for the HCoV-NL63 stock propagated in LLC-MK2 cells, which was used as an infectious dose for subsequent experiments described herein.

### Immunofluorescence and morphological analysis used for LLC-MK2 cells lack sensitivity to detect HCoV-NL63 infection in monolayer HREC or organotypic ALI-HREC cultures derived from human primary tracheobronchial epithelial cells.

**FIG 3:**
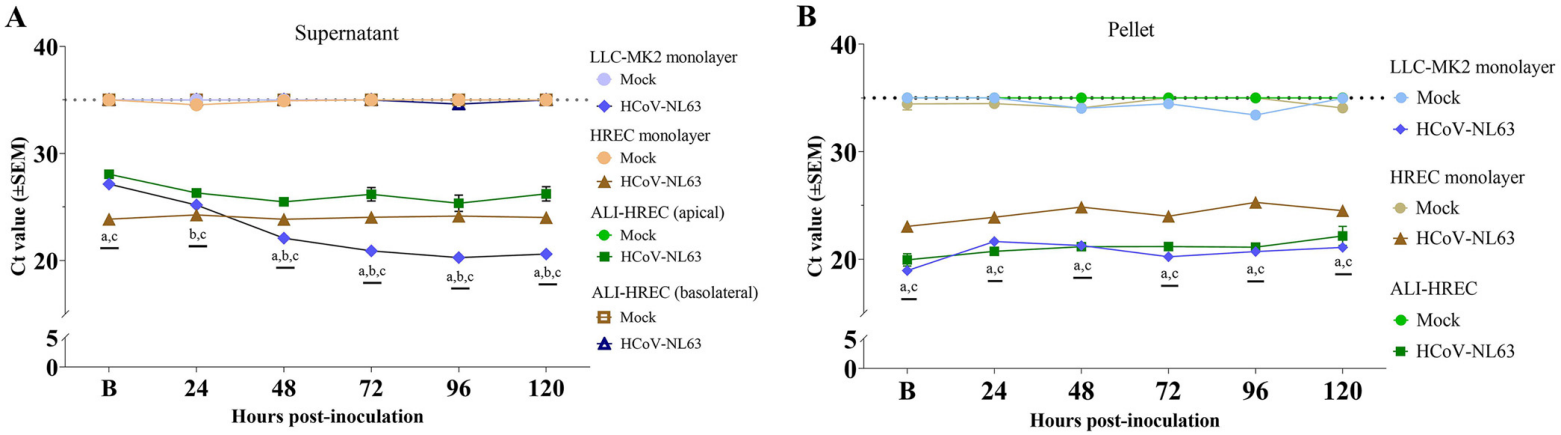
HCoV-NL63 growth kinetics in monolayer cultures of LLC-MK2 cells and primary human respiratory epithelial cells (HRECs), and organotypic airway cultures of human respiratory epithelial cells (ALI-HRECs) inoculated with a viral dose of 1 × 10^5^ TCID_50_/mL. The first time point (denoted as B) corresponded to the collection immediately after inoculum removal and subsequent washing step, i.e., 2 h postinoculation (hpi) for LLC-MK2 or HRECs, and 6 hpi for ALI-HRECs. Viral RNA detection was determined using real time quantitative PCR (RT-qPCR) and expressed as mean threshold cycle (*C_T_*) value (±standard error of the mean [SEM]). A *C_T_* value of 35 was used as cut-off. (A) Viral kinetic curve generated using HCoV-NL63 N gene-based RT-qPCR data from HCoV-NL63- (1 × 10^5^ TCID_50_/mL) or mock-inoculated culture supernatants (*n* = 3) of LLC-MK2 cells and HRECs, and basolateral media from ALI-HRECs. Additionally, a 100 μL LHC medium (Thermo Fisher Scientific) was added onto ALI-HRECs to also collect an apical wash for each time point. (B) Viral kinetic curve generated using cell pellets (*n* = 3) from LLC-MK2 cells, HRECs, and ALI-HRECs. Significant statistical differences (*P < *0.01) across virus-inoculated cultures are denoted with *a* [LLC-MK2 versus HREC], *b* [LLC-MK2 versus ALI-HREC], and *c* [HREC versus ALI-HREC] for each time point. No viral RNA amplification was detected at any time point in any of the mock-inoculated cultures (LLC-MK2, HRECs, and ALI-HRECs), being the *C_T_* values significantly different (*P < *0.0001) compared to the obtained for the virus-inoculated cultures throughout the study. However, for clarity, the significant differences are not denoted in the graph.

Confluent monolayer cultures of LLC-MK2 cells and HRECs, and ALI-HREC cultures inoculated with a high infectious dose (1 × 10^5^ TCID_50_/mL) of HCoV-NL63, were comparatively evaluated for the presence of virus-specific CPE and expression of HCoV-NL63 N protein by IFA over the course of 96 hpi (Fig. 3A). In contrast to the pattern observed for mock (Fig. 1A)- or virus-inoculated LLC-MK2 cells (Fig. 1D), HCoV-NL63 infection was not associated to any CPE or morphological change in monolayers of HREC cultures (Fig. 1E) compared to mock-inoculated control (Fig. 1B). Similarly, HCoV-NL63 did not disrupt the integrity of the pseudostratified epithelia (Fig. 1C, mock; Fig. 1F, infected) nor caused alteration on ciliary beating or sinchronicity in ALI-HREC cultures (Video S1) compared to mock-inoculated cultures (Video S2). HCoV-NL63 N protein expression, and therefore infection, was detected by IFA as early as 48 hpi only in virus-inoculated LLC-MK2 cultures. Contrary than for LLC-MK2 (Fig. 1G, mock; Fig. 1J, virus), the IFA was not sensitive enough to detect HCoV-NL63 N protein in monolayer HREC (Fig. 1H mock; Fig. 1K, virus) nor in ALI-HREC cultures (Fig. 1I, mock; Fig. 1L, virus).

### RT-qPCR analysis indicates higher HCoV-NL63 replication in LLC-MK2 cells compared to ALI-HRECs, and absence of virus replication in HRECs.

Viral kinetic curves were generated using HCoV-NL63 N gene-based RT-qPCR data (compiled in Table 1) using the supernatants collected from HCoV-NL63-inoculated (1 × 10^5^ TCID_50_/mL) or mock-inoculated of LLC-MK2 cells and HRECs and the basolateral/apical extracellular medium of ALI-HREC cultures (Fig. 3A). Results indicate a significant (*P < *0.05) virus replication in LLC-MK2 cells compared to HRECs and ALI-HRECs throughout the infection, with at least five *C_T_* reduction after 24 hpi. Moreover, a virus replication trend was detected only in the apical extracellular media of ALI-HREC-infected cultures, significantly different (*P < *0.01) with HRECs across the infection period. Interestingly, no viral RNA was detected in the basolateral medium collected from ALI-HREC cultures.

**TABLE 1:** Characterization of HCoV-NL63 infection across different culture systems^*a*^

Cell type/inoculation	24 hpi			48 hpi			72 hpi			96 hpi				
	*C_T_* value (±SEM)		CPE/IFA	*C_T_* value (±SEM)		CPE/IFA	*C_T_* value (±SEM)		CPE/IFA	*C_T_* value (±SEM)		CPE/IFA	*C_T_* value (±SEM)	
	Supernatant	Pellet		Supernatant	Pellet		Supernatant	Pellet		Supernatant	Pellet		Supernatant	Pellet
LLC-MK2														
Mock	35. (±0.0)	35.0 (±0.0)	−/−	35.0 (±0.0)	35.0 (±0.0)	−/−	35.0 (±0.0)	34.0 (±0.3)	−/−	35.0 (±0.0)	34.5 (±0.1)	−/−	35.0 (±0.0)	33.4 (±0.3)
HCoV-NL63	27.1 (±0.2)	18.9 (±0.1)	−/−	25.2 (± 0.1)	21.7 (±0.1)	−/+	22.1 (±0.0)	21.3 (±0.3)	+/+	20.9 (±0.1)	20.2 (±0.2)	+/+	20.3 (±0.1)	20.7 (±0.3)
HREC														
Mock	35.0 (±0.0)	34.4 (±0.6)	−/−	34.6 (±0.4)	34.5 (±0.3)	−/−	34.9 (±0.1)	34.1 (±0.4)	−/−	35.0 (±0.0)	35.0 (±0.0)	−/−	35.0 (±0.0)	35.0 (±0.0)
HCoV-NL63	23.9 (±0.2)	23.1 (±0.2)	−/−	24.3 (±0.1)	23.9 (±0.1)	−/−	23.8 (±0.1)	24.8 (±0.3)	−/−	24.0 (±0.2)	24.0 (±0.2)	−/−	24.1 (±0.1)	25.3 (±0.2)
ALI-HREC														
Mock														
Apical	35.0 (±0.0)	35.0 (±0.0)	−/−	35.0 (±0.0)	35.0 (±0.0	−/−	35.0 (±0.0)	35.0 (±0.0)	−/−	35.0 (±0.0)	35.0 (±0.0)	−/−	35.0 (±0.0)	35.0 (±0.0)
Basolateral	35.0 (±0.0)			35.0 (±0.0)			35.0 (±0.0)			35.0 (±0.0)			35.0 (±0.0)	
HCoV-NL63														
Apical	28.1 (±0.3)	19.9 (±0.6)	−/−	26.3 (±0.2)	20.7 (±0.2)	−/−	25.5 (±0.4)	21.2 (±0.2)	−/−	26.2 (±0.6)	21.2 (±0.0)	−/−	25.3 (±0.8	21.1 (±0.2)
Basolateral	35.0 (±0.0)			35.0 (±0.0)			35.0 (±0.0)			35.0 (±0.0)			34.6 (±0.4)	

a Cytopathic effects (CPE), indirect immunofluorescence assay (IFA), and real-time quantitative PCR (RT-qPCR) results were obtained for HCoV-NL63- (1 × 10^5^ TCID_50_/mL) or mock-inoculated monolayer cultures of LLC-MK2 cells, primary human respiratory epithelial cell culture (HREC), and organotypic airway HREC cultures (ALI-HRECs) monitored every 24 h after initial baseline sampling (B), for a total period of 96 h postinoculation (hpi). The first time point corresponded to the collection immediately after inoculum removal and subsequent washing step, i.e., 2 hpi for LLC-MK2 or HREC, and 6 hpi for ALI-HREC. The presence of CPE and HCoV-NL63 N protein-specific immunofluorescence signal is represented with a (+) sign, while the absence of infection is represented with a (−) sign. Viral RNA detection was determined using RT-qPCR and expressed as mean *C_T_* values (±SEM).

Likewise, the viral kinetic curves obtained from RT-qPCR data (Table 1) corresponding to cell pellets (*n* = 3) from LLC-MK2 cells, HRECs, and ALI-HRECs are presented in Fig. 3B The *C_T_* values from LLC-MK2 and ALI-HREC cell pellets were significantly lower (*P < *0.01) than the HREC cultures throughout the course of the infection. In contrast, no significant differences (*P > *0.05) on the *C_T_* values between cell pellets of LLC-MK2 and ALI-HRECs were found at any time point.

Noteworthy, no virus replication was detected in virus-inoculated HREC cultures regardless the culture fraction (Fig. 3A, supernatant; Fig. 3B, pellet) analyzed. Although as was the case for LLC-MK2 and ALI-HRECs, the *C_T_* values in HCoV-NL63-inoculated HRECs were significantly lower (*P < *0.0001) than the mock-inoculated controls for which, and as expected, no amplification was detected at any time point, these *C_T_* values remained constant over time (Fig. 3A, supernatant; Fig. 3B, pellet).

Based on the individual growth kinetic curves for individual cultures over time, significant virus replication was detected only in the supernatant of LLC-MK2 cells (within 48 hpi; *P < *0.001) and ALI-HRECs (within 24 hpi; *P < *0.01). No significant virus replication was detected on the pellet fraction of LLC-MK2, ALI-HREC, or HREC cultures.

### Comparative flow cytometric analysis reveal higher ACE2 protein expression and higher HCoV-NL63 infection rate in LLC-MK2 cells, followed by ALI-HRECs, and no significant ACE2 receptor protein and viral N protein expression in HRECs.

**FIG 4:**
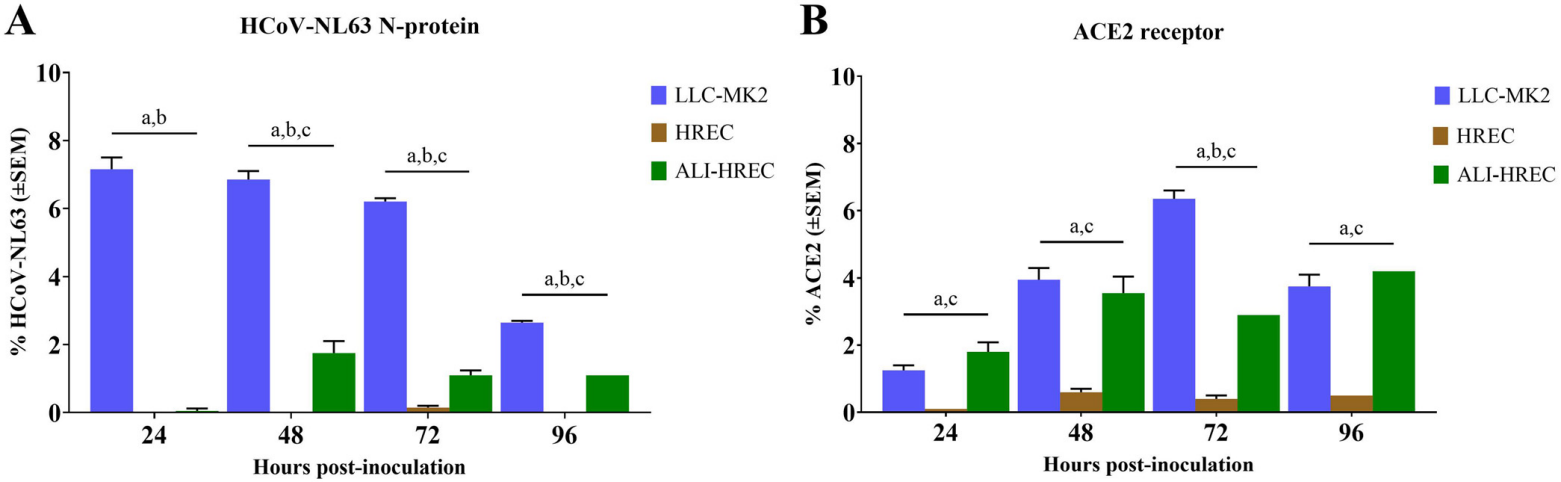
Flow cytometric analysis of subpopulations of LLC-MK2 cells, HRECs, and ALI-HRECs inoculated with HCoV-NL63 (1 × 10^5^ TCID_50_/mL) based on the relative expression of the HCoV-NL63 N protein (A) and the ACE2 receptor protein (B). Cell percentages are expressed as mean ± standard error of the mean (SEM). Statistically significant differences (*P < *0.01) are denoted with *a* [LLC-MK2 versus HREC], *b* [LLC-MK2 versus ALI-HREC], and *c* [HREC versus ALI-HREC] for each time point.

Flow cytometric analysis of the percentage of live cells expressing either or both the ACE2 receptor protein (ACE2+) and the HCoV-NL63 N protein (NL63+) revealed a significantly higher percentage of cells (>90%) across the different cultures (LLC-MK2, HREC, and ALI-HREC) showing a double negative ACE2-/NL63 phenotype, independently of virus (Fig. S3A)- versus mock-inoculation treatments (Fig. S3B). Particularly, comparative analyses upon specific subpopulations of cells expressing HCoV-NL63 N protein and ACE2 receptor protein that were collected over time (24, 48, 72, and 96 hpi) from LLC-MK2, HREC, and ALI-HREC cultures inoculated with same dose of HCoV-NL63 (1 × 10^5^ TCID_50_/mL) are presented in Fig. 4.

Overall, monolayers of LLC-MK2 cells showed significant higher (*P < *0.01) infection rate (% cells expressing viral N protein) compared to both HRECs and ALI-HRECs through the observational period (Fig. 4A). Likewise, pseudostratified cultures of HRECs growing in ALI were significantly (*P < *0.01) more susceptible to HCoV-NL63 infection than same cells growing in monolayer between 48 and 96 hpi.

In addition, flow cytometric results relative to ACE2 protein (Fig. 4B) indicated significantly higher (*P < *0.01) expression levels in both LLC-MK2 and ALI-HREC cultures compared to HREC throughout the study. However, the only significant difference between LLC-MK2 and ALI-HREC culture was found at 72 hpi, with higher ACE2 expression in LLC-MK2 cells. However, we did not find a significant correlation between viral N protein and ACE2 receptor protein expression across different cell cultures evaluated in the present study.

### Gene and protein expression analysis of ACE2 receptor on individual populations of LLC-MK2 cells, HRECs, and ALI-HRECs shows no significant modulation over the course of the infection.

**FIG 5:**
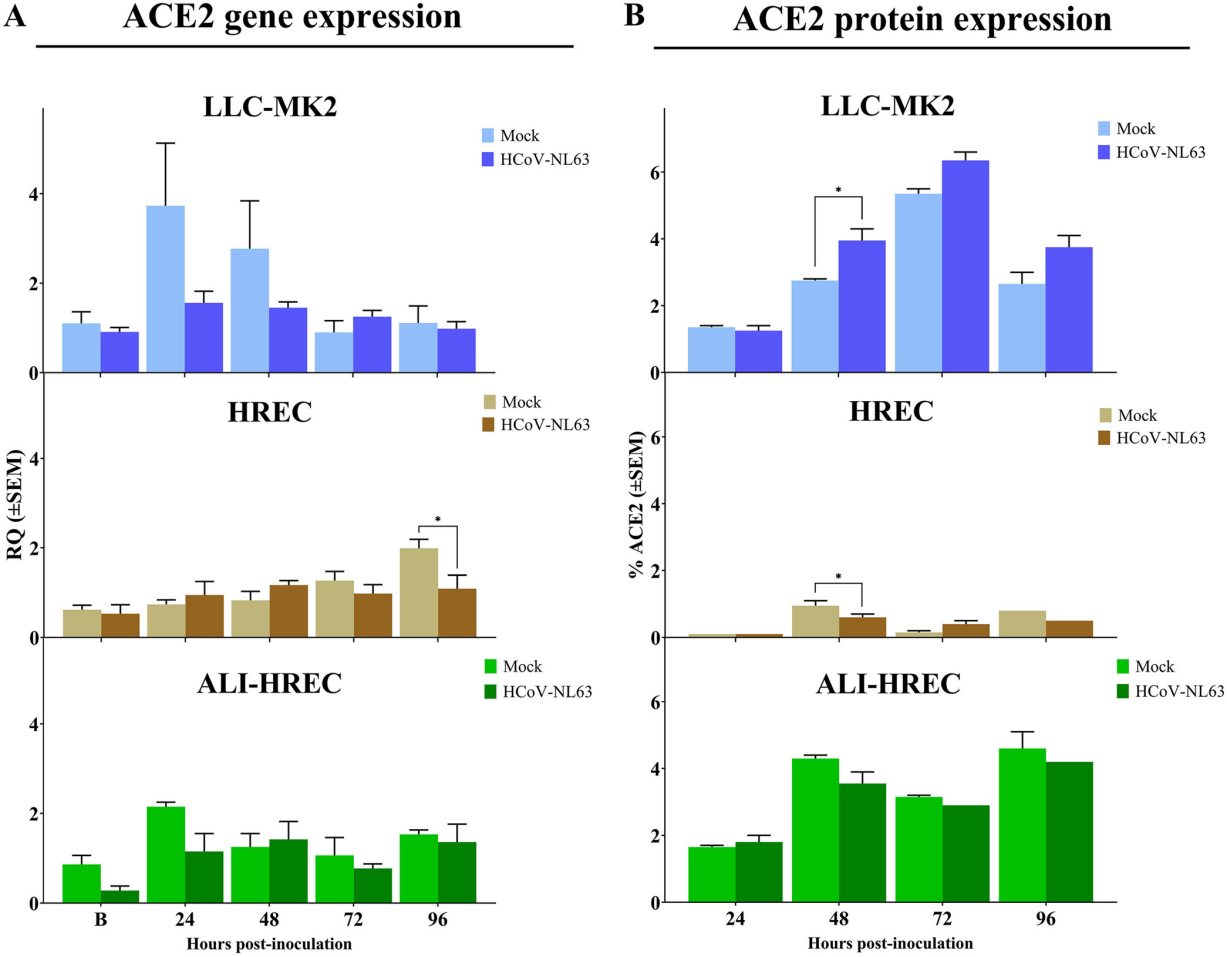
Effect of HCoV-NL63 infection on the expression of ACE2 receptor within each culture type or cell population, i.e., monolayer cultures of LLC-MK2 cells and HRECs, and organotypic ALI-HRECs. (A) Gene expression levels of angiotensin-converting enzyme 2 (ACE2) were determined by quantitative PCR (qPCR) and expressed as relative quantification (RQ) values (±SEM) for HCoV-NL63- and mock-inoculated cells. (B) Protein expression levels of ACE2 were determined by flow cytometry analysis and expressed as percentages (±SEM) for HCoV-NL63- and mock-inoculated cells. Expression levels were compared at 24, 48, 72, and 96 h postinoculation (hpi) for each culture type. * Statistically significant difference denoted with *P < *0.05.

A longitudinal analysis of the gene (RT-qPCR) (Fig. 5A) and protein (flow cytometry) expression (Fig. 5B) of ACE2 receptor within each population of LLC-MK2 cells, HRECs, and ALI-HRECs inoculated with HCoV-NL63 (1 × 10^5^ TCID_50_/mL) or mock inoculated with culture medium showed no significant modulation over time. The only punctual statistically significant difference (*P < *0.05) was found in LLC-MK2 cells with higher ACE2 protein expression at 48 hpi in HCoV-NL63 inoculated cells, but HRECs showed lower ACE2 gene expression at 96 hpi and lower protein expression at 48 hpi in HCoV-NL63-inoculated group.

## DISCUSSION

HCoV-NL63 is an important contributor to upper and lower respiratory tract infections mainly in children, but also in adults (3). Different strategies have been proposed to suppress HCoV-NL63 replication by either targeting the virus or enhancing host antiviral mechanisms (G. Castillo, J. C. Mora-Díaz, M. Breuer, P. Singh, R. K. Nelli, and L. G. Giménez-Lirola, submitted for publication). Often, preclinical antiviral screening relies on the detection and quantification of replicating viruses in cell culture. Moreover, cell culture remains the gold standard for virus isolation and laboratory differential diagnosis of viral respiratory diseases (20).

HCoV-NL63 establishes infection primarily in the mucosal epithelia of the upper respiratory tract of humans (21). This study evaluated possible differences on the susceptibility and virus replication efficiency of submerged monolayer cultures of LLC-MK2 and HRECs and organotypic ALI-HRECs infected with HCoV-NL63. LLC-MK2 cells, established initially from a pooled cell suspension prepared from the kidney tissue of six adult rhesus monkeys (Macaca mulatta) in the mid-1950s (22), were used as a reference because these cells allow virus replication at high titers and are commonly used for HCoV-NL63 isolation and propagation from clinical specimens (2, 11, 12). The present study provided complete protocols for virus culture and propagation of LLC-MK2, HREC, and ALI-HREC cultures, including specific culture medium and supplements, antibiotic mix to prevent bacteria contamination, and optimal culture conditions.

In general, HCoV-NL63 rarely shows CPE (11). In line with previous reports (13, 23), we observed subtle to weak or diffuse and transient CPE on LLC-MK2 cells only at the higher infectious doses (>1 × 10^3^ TCID_50_/mL) assesed in this study. Contrary, HCoV-NL63 infection was not associated with any CPE or morphological changes in monolayers of HREC cultures and did not disrupt the ciliated pseudostratified columnar epithelia of ALI-PREC cultures, maintaining its integrity (absence of CPE or viral RNA in the basolaterial extracellular medium) and cilia synchronicity, nor did it cause an alteration on ciliary beating in organotypic ALI-HREC cultures. Our study confirms that the TCID_50_ or plaque assays based on CPE observation are not applicable to demonstrate virus viability nor for infection or for virus titration experiments across different cell types or culture models.

The use of monoclonal antibodies for immunofluorescence assay and the molecular amplification of viral RNA by PCR allow sensitive and accurate detection of HCoV-NL63 infection and replication regardless of the presence or absence of CPE or earlier before its appearance (11, 24). We detected HCoV-NL63 infection by IFA only in LLC-MK2 cells, but this technique was not sensitive enough to detect the low levels of virus infection otherwise detected in HREC monolayers and organotypic ALI-HREC cultures by RT-qPCR and flow cytometry assays. The sgRNA coding N protein is the most abundant RNA detected in infected LLC-MK2 cells and the structural protein more abundantly expressing during the infection (25), which justify the use of N gene and N protein as infection-verification target for RT-qPCR and flow cytometry, respectively.

HCoV-NL63 enter respiratory epithelial cells, its primary target for infection and initial replication, via the ACE2 receptor (19). The RT-qPCR and flow cytometric results from this study demonstrated higher susceptibility to HCoV-NL63 infection and virus replication in LLC-MK2 cells followed by ALI-HRECs, with very low susceptibility and no significant virus replication in HRECs. Overall, both LLC-MK2 and ALI-HRECs expressed more ACE2 receptors than HRECs, with no significant differences (except at 72 hpi) between the percentage of LLC-MK2 and ALI-HRECs expressing ACE2 protein. Although the differences on the susceptibility to HCoV-NL63 could be associated with the relative expression of ACE2 receptors across the different types of cultures evaluated in this study, we did not find significant correlation in the present study. Therefore, a more refined *in vitro* culture model should be based on the isolation and subculture for expansion of a specific subpopulation of ACE2-expressing cells.

Human respiratory cells grown in air-liquid interface as organotypic airway cultures resemble morphologically (basal, ciliated, and secretory cells) and functionally (beating cilia and mucus secretion) the human airway (26). In line with our results, Sungnak et al. (27) observed a higher expression levels of the receptor ACE2 in both goblet cells and ciliated cells. Although relatively easy to maintain, monolayer cultures of primary respiratory epithelial cells lack the complexity of organotypic ALI-HREC cultures and expressed significantly less ACE2 receptors than ALI-HRECs, which correlated with the lack of HCoV-NL63 replication in HRECs. Indeed, ALI-HREC cultures have been successfully used to investigate different HCoVs, including HCoV-NL63 (14, 28, 29), HCoV-229E (29, 30), HCoV-HKU1 (29, 31), HCoV-OC43 (29, 32), SARS-CoV (33), Middle East respiratory syndrome-CoV (MERS-CoV) (34), and SARS-CoV-2 (17).

The present study demonstrates that ACE2 receptor is a critical factor for susceptibility to HCoV-NL63 infection and replication, as is the type of culture used during infection studies. In particular, more refined *in vitro* culture models should be based on organotypic cultures derived from subpopulations of ACE2 expressing human primary respiratory epithelial cells that recapitulate the proxy of cell composition, structure, and functionality of the respiratory epithelia *in vivo*. These cultures have broadened virological studies in the human context, particularly in recent uses for research in emerging coronaviruses.

## MATERIALS AND METHODS

### Cell cultures.

**(i) LLC-MK2 cells monolayer culture.** Rhesus monkey (Macaca mulatta) kidney epithelial cells (LLC-MK2 cells) (CCL-7; ATCC, Manassas, VA) were cultured using growth medium #1 (Eagle’s minimum essential medium [ATCC] supplemented with heat-inactivated 10% fetal bovine serum [FBS; ATCC], 100 IU/mL penicillin, 100 μg/mL streptomycin [Gibco, Thermo Fisher Scientific, Waltham, MA, USA], and 25 μg/mL gentamicin [Gibco, Thermo Fisher Scientific]) and incubated at 37°C with 5% CO_2_.

**(ii) Human respiratory epithelial cells monolayer culture.** HRECs (bronchial/tracheal epithelial cells; PCS-300-010; ATCC) were cultured in growth medium #2 (airway epithelial cell basal medium [PCS-300-03; ATCC] supplemented with bronchial epithelial cell growth kit [PCS-300-040; ATCC], 100 IU/mL penicillin, 100 μg/mL streptomycin, and 2.5 μg/mL amphotericin B [Corning, Corning, NY, USA]) and incubated at 37°C with 5% CO_2_.

**(iii) Air-liquid interface organotypic airway culture.** ThinCert cell culture inserts (Greiner Bio-one, Monroe, NC, USA), 0.4-μm pore size, for 24-well plates were incubated with bovine collagen I/III solution (Purecol Solution; 40 μg/mL; Advanced Biomatrix, Carlsbad, CA, USA) for 24 h at 37°C or until they became dried. On the day of cell seeding, cell culture inserts were exposed to UV light for 15 min, and then both sides of the membrane were washed with phosphate-buffered saline (PBS). Between 0.8 × 10^5^ and 1.6 × 10^5^ HRECs resuspended in 100 μL of growth medium #2 were seeded in an insert, followed by the addition of 500 μL of the same medium into the platewell. Plates were incubated at 37°C with 5% CO_2_ for 24 h; thereafter, medium was removed, and wells were rinsed and replaced with growth medium #2 supplemented with 0.1 μM retinoic acid (Acros Organics, Carlsbad, CA, USA), on both sides of the membrane. After the first day, the medium from top of the membrane was removed so the medium was only present at the bottom in the platewell creating the air-liquid interface. Any medium from the top surface was removed daily until the membrane remained visibly dry around 3 to 6 days after seeding. Thereafter, the medium was changed every other day until ~28 days under air-liquid to promote cell differentiation (Fig. S1). Upon complete differentiation and besides normal daily fluctuations, transendothelial electrical resistance values of ALI-HREC cultures remained stable (250 to 350 Ω/cm2).

### Virus propagation and titration.

HCoV-NL63 (the following reagent was deposited by the Centers for Disease Control and Prevention and obtained through BEI Resources, NIAID, NIH: Human Coronavirus, NL63, NR-470) was propagated in LLC-MK2 cells in 175-cm^2^ tissue culture flasks (Nunc EasYFlask; Thermo Fisher Scientific) incubated at 37°C with 5% CO_2_. After 23 h (>90% cell confluence), flasks were transferred to a 34°C incubator with 5% CO_2_ for 1 h to allow cell adaptation prior to virus inoculation (HCoV-NL63 grows slightly better at 34°C in MK2 cells) The growth medium #1 was decanted, and cells were rinsed twice with PBS pH 7.4 (Gibco, Thermo Fisher Scientific). Cells were inoculated with HCoV-NL63 at 1 × 10^5^ 50% tissue culture infectious dose per mL (TCID_50_/mL), and incubated at 34°C with 5% CO_2_ for 1.5 h. After that, the inoculum was removed, and the infection medium #1 (growth medium without FBS) was added to the flask and incubated for 5 days at 34°C with 5% CO_2_. The virus was harvested through three −80°C freeze/thaw cycles, followed by removing cellular debris by centrifugation at 3,000 × *g* for 20 min at 4°C in 50 mL conical tubes (Corning). The supernatant was collected, aliquoted into 2-mL cryovials (Corning), and stored at −80°C to be used as virus stock.

Virus titration was performed on a 96-well clear flat bottom, black polystyrene surface-treated microplate (CellBIND Costar; Corning) seeded with 1 × 10^4^ LLC-MK2 cells per well with growth medium #1 and incubated at 37°C with 5% CO_2_. As previously described, the microplate was transferred to a 34°C incubator with 5% CO_2_ prior to infection. After 1 h of temperature adaptation, cells were rinsed twice with 100 μL of PBS. In seven columns of a dilution plate, 12 10-fold serial dilutions (10^−1^ to 10^−12^) of virus stock were performed using infection medium #1. Subsequently, 100 μL of each virus dilution were transferred to the test plate and incubated at 34°C with 5% CO_2_. One column was mock inoculated with infection medium #1 and served as a negative control. After 5 days postinoculation, cells were fixed with 80% acetone for 15 min at room temperature. Cells were inoculated with 100 μL per well of mouse anti-HCoV-NL63 nucleocapsid (N) protein monoclonal antibody (2D4; Ingenasa-Eurofins, Madrid Spain) (0.25 μg/mL) diluted in PBS with 0.1% bovine serum albumin (BSA; Jackson ImmunoResearch Inc., West Grove, PA USA), and incubated at 37°C for 1 h. After washing three times with 200 μL of PBS, 50 μL of fluorescein isothiocyanate (FITC)-conjugated goat anti-mouse antibody IgG (KPL; SeraCare Life Sciences Inc., Mildford, MA, USA) (20 μg/mL) diluted in PBS with 0.1% BSA was added to each well and incubated at 37°C for 45 min. Plates were then washed three times with 200 μL of PBS per well, followed by the addition of 100 μL of PBS before reading on an inverted microscope (Olympus CKX4 microscope; Olympus Olympus Corp., Center Valley, PA, USA) and Infinity 2 camera, and Infinity Analyze imaging software (Ver 6.5.5; Lumenera Corp, Ottawa, ON, Canada). A well was classified as HCoV-NL63-positive if the presence of specific staining was evident. Virus titers were calculated using the Spearman-Karber method (35) and expressed as TCID_50_ per millilter.

### HCoV-NL63 N gene-based RT-qPCR.

Viral RNA extractions were performed using the E.Z.N.A Viral RNA Kit (Omega Bio-tek, Inc., Norcross, GA, USA) and vacuum manifold (Qiagen, Germantown, MD, USA) method following the manufacturer’s instructions. An adapted quantitative HCoV-NL63 N gene-based reverse transcription-PCR (RT-qPCR) (11) was used to confirm and quantify HCoV-NL63 *in vitro* infection on monolayers of LLC-MK2 cells and HRECs, and organotypic ALI-HREC culture. Each RT-qPCR (20 μL final reaction volume) was set up by combining 3 μL of the extracted sample RNA, 5 μL of 4× TaqPath 1-Step RT-qPCR Master Mix, CG (Applied Biosystems, Thermo Fisher Scientific, Foster City, CA, USA), 0.8 μL of 10 μM (each) forward primer 5′-GCGTGTTCCTACCAGAGAGGA-3′ and reverse primer, 5′-GCTGTGGAAAACCTTTGGCA-3′, and 0.2 μL of the 10 μM probe 5′-FAM-ATGTTATTCAGTGCTTTGGTCCTCGTGAT-BHQ1-3′. All RT-qPCRs were performed in triplicates, with a positive control (the following reagent was obtained through BEI Resources, NIAID, NIH: Genomic RNA from Human Coronavirus [HCoV], NL63, NR-44105) and a “no template” control (NTC) included in each run. RT-qPCRs were run on a Rotor-Gene Q (Qiagen) with cycling conditions, holding of 25°C for 2 min Uracil *N*-glycosylase, 50°C for 15 min reverse transcription and 95°C for 2 min enzyme activation; followed by 40 cycles, 95°C for 3 s denaturation, and 60°C for 30 s amplification. The RT-qPCR results were analyzed using Rotor-Gene Q–Pure Detection software (v 2.3.1), samples with threshold cycle (*C_T_*) above 35 were considered negative. Subsequently the data exported to Microsoft Excel and GraphPad Prism 9.2.0 software (GraphPad Software Inc., La Jolla, CA, USA) for further analysis.

### HCoV-NL63 growth kinetics.

**(i) Monolayer LLC-MK2 cells culture.** HCoV-NL63 growth kinetics in LLC-MK2 cells were performed by seeding 3.5 × 10^4^ cells per well in 24-well plates (Greiner Bio-one) using growth medium #1 and incubated at 37°C with 5% CO_2_. After 23 h, plates were transferred to a 34°C incubator prior to infection. One hour after cell temperature adaptation, growth medium #1 was decanted and cells were rinsed twice with 500 μL of PBS. LLC-MK2 cells were inoculated by triplicate with two different doses of HCoV-NL63 (1 × 10^5^ TCID_50_ per mL) or mock inoculated with infection medium #1 and incubated for at 34°C with 5% CO_2_. After 2 h postinoculation (hpi) cells were washed twice with PBS, fresh infection medium #1 was added to each well, and plates were incubated at 34°C with 5% CO_2_ for 96 hpi.

**(ii) Monolayer HREC culture.** Viral kinetics in HRECs were performed by seeding 5 × 10^4^ cells per well in collagen-coated 24-well plates (Greiner Bio-one) using growth medium #2. After 24 h of incubation, cells were washed twice with 500 μL of LHC base medium (Gibco, Thermo Fisher Scientific), and inoculated by triplicate with 1 × 10^5^ TCID_50_ per mL of HCoV-NL63 or mock inoculated with infection medium #2 (airway epithelial cell basal medium [PCS-300-030; ATCC] supplemented with 2% Ultroser G [Sartorius, Göttingen Germany], 1% MEM nonessential amino acids solution [Gibco, Thermo Fisher Scientific], 1% HEPES [Gibco, Thermo Fisher Scientific], 1% GlutaMax [Gibco, Thermo Fisher Scientific], 100 IU/mL penicillin, and 100 μg/mL streptomycin) and incubated at 37°C with 5% CO_2_ for 2 h. Cells were rinsed with LHC basal medium, fresh infection medium #2 was added to each well, and plates were incubated at 37°C with 5% CO_2_ for 96 h.

**(iii) Organotypic ALI-HREC culture.** Viral kinetics in ALI-HRECs were performed in HRECs grown in ThinCert cell culture inserts using growth medium #2 supplemented with 0.1 μM retinoic acid (Acros Organics). Before virus inoculation, cells were washed twice with 200 μL of LHC base medium (Gibco, Thermo Fisher Scientific) and inoculated (apial side) by triplicate with 1 × 10^5^ TCID_50_/mL of HCoV-NL63 or mock inoculated with growth medium #2 supplemented with 0.1 μM retinoic acid (Acros Organics), and incubated at 37°C with 5% CO_2_ for 6 h. Cells were rinsed with LHC basal medium, fresh growth medium #2 supplemented with 0.1 μM retinoic acid (Acros Organics) was added to each well, and plates were incubated at 37°C with 5% CO_2_ for 96 hpi.

Every 24 h (24, 48, 72, and 96 hpi), LLC-MK2 cells and HREC monolayer cultures and organotypic ALI-HREC cultures were observed under inverted microscope to determine possible presence of CPE, supernatants were also taken from each culture for RT-qPCR analysis, and corresponding plates were fixed with 300 μL per well of 2.5% glutaraldehyde (Electron Microscopy Sciences, Hatfield, PA, USA) for 20 min at room temperature for IFA.

### Indirect immunofluorescence assay.

Fixed HCoV-NL63-infected LLC-MK2 cells and HRECs were washed three times with 500 μL of PBS. Each well was inoculated with 300 μL of 0.1% Triton in PBS with 0.1% BSA and incubated for 10 min at room temperature. After two washes with 500 μL of PBS, wells were inoculated with 500 μL of mouse anti-HCoV-NL63 N protein monoclonal antibody (2D4; Ingenasa-Eurofins) (0.25 μg/mL) diluted in PBS with 0.1% BSA, and incubated at 37°C for 1 h. After rising the wells three times with 500 μL of PBS, FITC-conjugated goat anti-mouse antibody IgG (KPL; SeraCare Life Sciences Inc.; 20 μg/mL) diluted 1:100 in PBS with 0.1% BSA was added to each well (500 μL), and plates were incubated at 37°C for 45 min. Plates were washed three times as indicated above, then 2 drops per mL of 4′,6-diamidino-2-phenylindole (DAPI; NucBlue, Invitrogen, Thermo Fisher Scientific) were added to the wells, and incubated at room temperature for 5 min. After a three washes step with PBS followed by the addition of 500 μL of PBS, plates were immediately read on an inverted microscope (Olympus CKX41: Olympus Corporation) using Infinity 2 camera operated with Infinity analyze V.6.5 software (Teledyne Lumenera, Ottawa, ON, Canada). A well was classified as HCoV-NL63 positive (infected) if the presence of specific fluorescence against viral N protein staining was detected.

### Flow cytometry.

To evaluate the characterization of HCoV-NL63 infection at a cellular level, LLC-MK2 cells (8 × 10^4^ cells per well) and HRECs (1.6 × 10^5^ cells per well) were seeded into 12-well plates (Greiner Bio-one) and propagated as previously described. Once confluent (>90%), both LLC-MK2 cells and HREC monolayers and organotypic ALI-HREC cultures were inoculated with HCoV-NL63 or mock-inoculated with infection medium (negative control) as previously described. Then, cells were collected using TrypLE Express enzyme (Thermo Fisher Scientific) and inactivated with corresponding cell-type growth medium supplemented with 10% FBS (EquaFETAL; Atlas Biologicals, CO, USA). Cells were washed with PBS and incubated in PBS containing 100 μg/mL Bovine Dnase I (MilliporeSigma, St. Loius, MO, USA) with 5 mM magnesium chloride (MilliporeSigma) for 15 min at room temperature. Following incubation, cell suspensions were passed through a 100-μm cell strainer (Greiner Bio-One), and cells were washed thoroughly by centrifuging at 200 × *g* for 5 min. Cell pellets (from duplicates for LLC-MK2 or HRECs, or triplicates for organotypic ALI-HRECs) were resuspended in 100 μL PBS, transferred into a well from a 96-well plate, and incubated for 15 min on ice. The remaining cells from other replicates (8 wells for LLC-MK2 or HRECs or 24 inserts from organotypic ALI-HRECs) were used for unstained, fluorescence minus one, and isotype controls.

To identify dead cells in our analysis, the cells were resuspended in 100 μL per well LIVE/DEAD Fixable Near-IR Dead Cell Stain Kit (Invitrogen, Thermo Fisher Scientific) diluted 1:200 with PBS. Cells for ACE2 staining were incubated 30 min on ice with 100 μL goat anti-ACE2 Alexa Fluor 488-conjugated antibody (4 μg/m;, R&D systems, Minneapolis, MN, USA) diluted 1:50 in FACS buffer (PBS, 0.5 to 1% BSA or 5 to 10% FBS, 0.1% NaN_3_ sodium azide), washed twice with 150 μL cold FACS buffer, and fixed with 100 μL BD Cytofix/Cytoperm solution for 20 min on ice. Afterwards, cells were washed twice with 150 μL cold Perm/Wash buffer followed by incubation with 100 μL cold Perm/Wash buffer to permeabilize it for 15 min on ice. Then, Pan-Cytokeratin or HCoV-NL63 staining were performed incubating with 100 μL cold Perm/Wash buffer (Biosciences) containing mouse anti-Pan-Cytokeratin (AE1/AE3) (Bio-Rad Laboratories, Hercules, CA, USA) diluted 1:2,000 with Perm/Wash buffer, or mouse monoclonal anti-HCoV-L63 N protein (1 μg/mL; 2D4; Ingenasa-Eurofins) for 30 min on ice. Then, cells were washed twice with 150 μL cold Perm/Wash buffer followed by incubation with 100 μL goat anti-mouse Alexa Fluor 647 (15 μg/mL; Jackson ImmunoResearch Laboratories, Inc.) diluted 1:100 with cold Perm/Wash buffer. After a 30-min incubation on ice, cells were washed twice with 150 μL cold Perm/Wash buffer, and each well was resuspended into 200 μL FACS buffer and transferred to a 1.5-mL Eppendorf tube for reading in the flow cytometer (Attune NxT; Thermo Fisher Scientific). Flow cytometry-gating strategy for the analysis of the expresion of ACE2 receptor protein and viral N protein is shown in Fig. S2.

### RNA extraction and cDNA synthesis.

Total RNA extractions from monolayers of LLC-MK2 cells and HREC monolayer, and organotypic ALI-HRECs were performed with TRIzol reagent (Thermo Fisher Scientific) using the E.Z.N.A. Total RNA Kit (Omega Bio-tek, Inc., Norcross, GA USA) and vacuum manifold (Qiagen) method following the manufacturer’s instructions. Eluted RNA was quantified using a NanoDrop one microvolume UV-Vis spectrophotometer (Thermo Fisher Scientific). Samples with A260/280 between 1.96 and 2.05 were used for cDNA synthesis from 40 ng of total RNA per sample using qScript XLT cDNA SuperMix kit (QuantaBio, Beverly, MA, USA) and then stored at −20°C.

### ACE2 gene expression.

Expression level of ACE2 receptor gene were evaluated by real-time quantitative PCR (RT-qPCR) using PerfeCTa qPCR ToughMix (QuantaBio) using specific primers for Macaca mulatta (NM_001135696) and Homo sapiens (NM_021804) ACE2. A set of three reference genes (18S, ACTB, and EIF3K) was used to normalize data. The specific sequences of the primers used were as follows: forward primer 5′-GAACCTGTGCCCCATGATG-3′, and reverse primer 5′-GAGTAATCATTAGAAACATGGAACAGAGA-3′for ACE2; forward primer 5′-TGCCAAGAATGTTTTCATTAATCAAG-3′, and reverse primer 5′-CGCCAGTCGGCATCGT-3′for 18S; forward primer 5′-GTCCGCCTAGAAGCATTTGC-3′, and reverse primer 5′-GGCCTCGCTGTCCACCTT-3′for ACTB; and forward primer 5′-ACCAGTTCAACCCAGCCTTCT-3′, and reverse primer 5′-TGAGGGCCTTCAGCAGGAT-3′for EIF3K. All RT-qPCRs were performed on a Rotor-gene Q system (Qiagen) using 1× PerfeCTa qPCR ToughMix (QuantaBio), 400 nM specific primers, and 1 ng (cells) of total RNA converted to cDNA. The cycling conditions used were as follows: 95°C for 30 s holding; 40 cycles, 95°C for 5 s denaturation and 60°C for 30 s amplification; final melting curve analysis was performed at 95°C for 15 s, 60°C for 1 min, and 95°C for 15 s. All RT-qPCRs were performed in duplicate, and NTCs were included in each batch. For relative quantification of ACE2 expression, gene expression data were normalized using the geometric mean of the three reference genes (36), and the relative quantification values were obtained using the ΔΔ*C_T_* method as previously described (37).

### Statistical analysis.

To determine the significance of the results, a multiple unpaired *t* test with Welch correction was used to compare differences between two groups, and Tukey’s multiple-comparison test for multiple groups. Prism 9.2.0 software (GraphPad Software, Inc.) was used for the statistical analysis. Values are expressed as mean (±SEM) of at least three replicates unless otherwise noted. Statistical significance was considered with *P < *0.05.
