# Supplementary material for: Human Air-Liquid-Interface Organotypic Airway Cultures Express Significantly More ACE2 Receptor Protein and Are More Susceptible to HCoV-NL63 Infection than Monolayer Cultures of Primary Respiratory Epithelial Cells
Source: Microbiol Spectr. 2022 Jul 12;10(4):e01639-22. doi: 10.1128/spectrum.01639-22 (PMC9431431; doi:10.1128/spectrum.01639-22)
Supplement: Supplemental file 1 — Supplemental material. Download spectrum.01639-22-s0003.pdf, PDF file, 0.8 MB [file spectrum.01639-22-s0003.pdf]

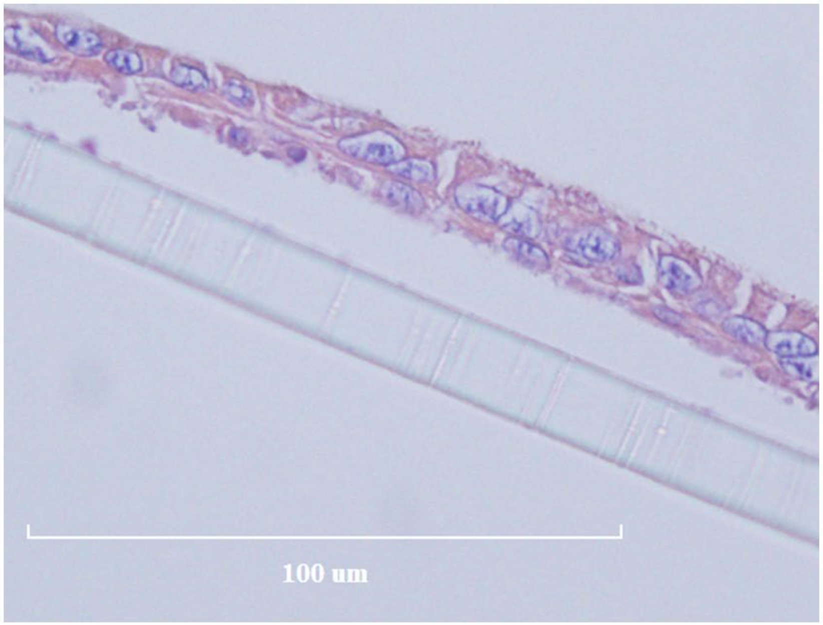

Fig. S1. H&E stained ALI-HREC culture.

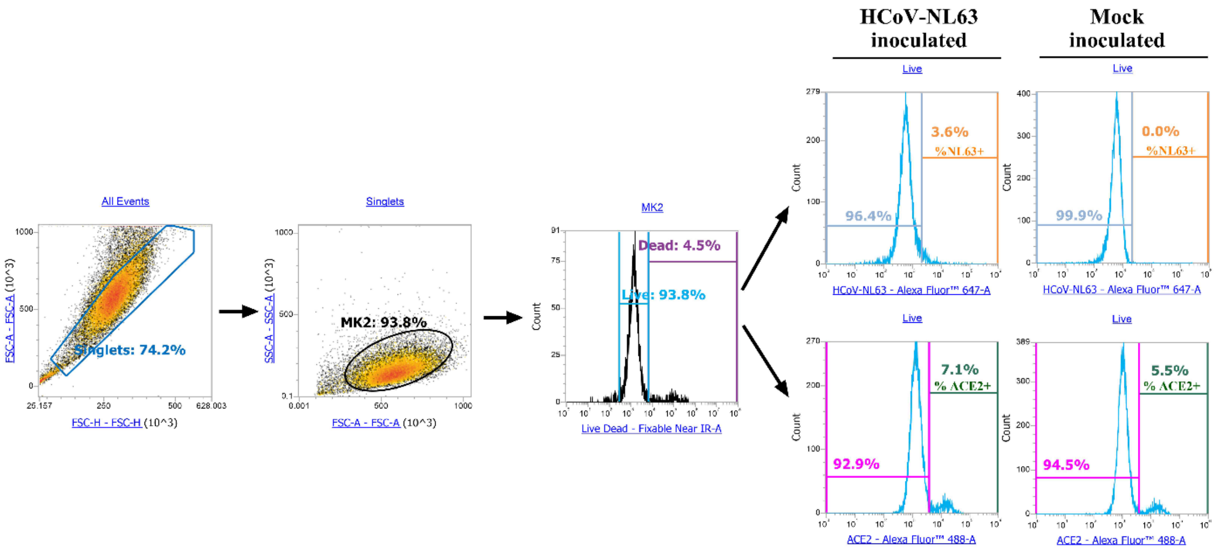

Fig. S2. Flow cytometry-gating strategy for the analysis of the expression of ACE2 receptor protein and HCoV-NL63 N-protein in monolayers of LLC-MK2 cells and human respiratory epithelial cells (HREC), and organotypic cultures of HREC (ALI-HREC). LLC-MK2 cells, HREC, and ALI-HREC were stained with LIVE/DEAD™ Fixable Near-IR Dead Cell Stain Kit (Invitrogen, Thermo Fisher Scientific), polyclonal goat anti-ACE2 conjugated with Alexa Fluor® 488 at a final concentration of 4 µg/mL (R&D Systems), IgG1 mouse monoclonal anti-HCoV-NL63 N protein (2D4, Ingenasa-Eurofins) at a final concentration of 1 µg/mL, and anti-mouse Alexa Fluor® 647 secondary antibody at a final concentration of 15 µg/mL (Jackson ImmunoResearch) and analyzed in the Attune™ NxT flow cytometer (Thermo Fisher Scientific). Each cell type was gated on FSC-A vs FSC-H plot to sort out the singlets population which were then gated in SSC-A vs. FSC-A density plot to exclude cell debris. Within each cell population (LLC-MK2, HREC, or ALI-HREC), live cells were gated with a histogram based on LIVE/DEAD™ Fixable Near-IR Dead cell staining to exclude dead cells from the analysis. The percentage of cells expressing HCoV-NL63 N protein (%NL63+) and ACE2 receptor protein (%ACE2+) were determine based on histograms using at least 10,000 cells from the live gate. FSC-H: forward scatter height; FSC-A: forward scatter area; SSC-A: side scatter area.

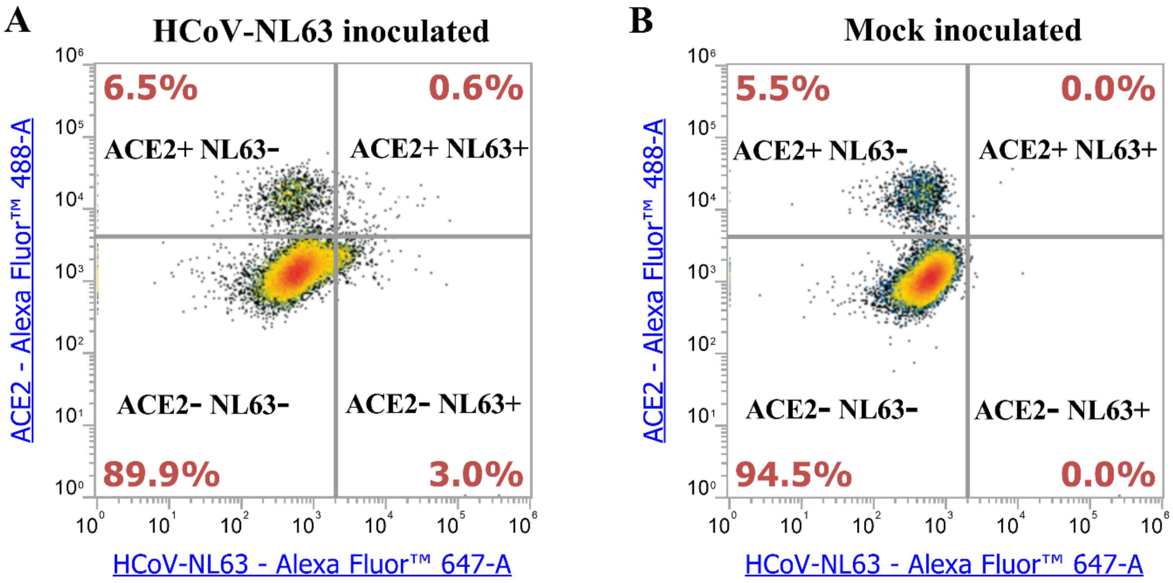

Fig. S3. Flow cytometry density plot showing the percentage of cells showing expression of the ACE2 receptor protein (Alexa Fluor® 488-A) and/or HCoV-NL63 N protein (Alexa Fluor® 647-A) in (A) HCoV-NL63-inoculated, and (B) Mock-inoculated cultures. A quadrant gate was used to determine the overall percentages of four different cell populations or phenotypes (ACE2+ NL63+, ACE2- NL63-, ACE2+ NL63-, ACE2- NL63+) for each cell type.

Video S1. Cilia motility in HCoV-NL63 inoculated ALI-HREC cultures.

Video S2. Cilia motility in mock-inoculated (culture medium) ALI-HREC cultures.
